# Retrospective Analysis of the Relationship between Two Anthrax Outbreaks in Kazakhstan Based on Genomic Data

**DOI:** 10.1128/MRA.01126-20

**Published:** 2020-12-10

**Authors:** Alexandr Shevtsov, Gilles Vergnaud, Asylulan Amirgazin, Larissa Lukhnova, Uinkul Izbanova, Vladislav Shevtsov, Yerlan Ramankulov

**Affiliations:** a National Center for Biotechnology, Nur-Sultan, Kazakhstan; b Université Paris-Saclay, Institute for Integrative Biology of the Cell (I2BC), Gif-sur-Yvette, France; c M. Aikimbayev’s National Scientific Center for Especially Dangerous Infections, Almaty, Kazakhstan; d School of Science and Technology, Nazarbayev University, Nur-Sultan, Kazakhstan; University of Maryland School of Medicine

## Abstract

We present a retrospective analysis of strains from two anthrax outbreaks in western Kazakhstan in 2009. The outbreaks occurred during the same period and in the same area located close to main roads, favoring a single source of infection. However, multilocus variable-number tandem-repeat analysis (MLVA), canonical single-nucleotide polymorphism (CanSNP) analysis, and genome-wide analysis demonstrated that the outbreaks were not connected.

## ANNOUNCEMENT

In June 2009, anthrax outbreaks occurred in two districts (Bayterek and Borili) of the West Kazakhstan region. The distance between the outbreaks is 118 km, and both locations are close to the main roads, suggesting the possibility of carryover contamination between the Bayterek and Borili districts (1). In both outbreaks, the sources of human infection were cows slaughtered without a veterinary examination. Eight strains were recovered from the Borili outbreak, from cattle or humans (one strain). Nine strains were recovered from the Bayterek outbreak, from the soil of the slaughter place or from clinical samples (five strains). Strains isolated from humans were anonymized, and their use was approved by the local ethics committee of the National Center for Biotechnology (protocol no. 1 from 1 January 2020). Strains were isolated using the inoculation method, plated on Hottinger agar, and cultivated at 37°C for 24 hours. All strains were identified as Bacillus anthracis based on the cultural and morphological characteristics of the colonies, the absence of hemolysis, catalase, lipase, and phosphatase, and protease activity, as well as their susceptibility to specific γ-phages. DNA was isolated from inactivated cultures using the QIAamp DNA minikit (Qiagen, USA) as previously described (2). Work with pathogens was performed in M. Aikimbayev’s National Scientific Center for Especially Dangerous Infections (NSCEDI) biosafety level 3 (BSL3) facilities.

Multilocus variable-number tandem-repeat analysis (MLVA) genotyping was performed *in vitro* with capillary electrophoresis of PCR amplicons using 31 variable-number tandem-repeat (VNTR) loci as previously described (2–4). Two genotypes were observed, genotype 4-20-14-57-21-1-9-7-13-30-7-30-45-10-16-11-11-13-14-75-64-9-8-9-8-6-20-5-5-4-4, shared by all strains from Borili, and genotype 4-20-14-53-17-2-7-9-16-26-7-70-24-10-16-11-11-13-14-57-64-11-8-9-8-6-20-4-4-4-5, shared by all strains from Baytirek. According to the MLVA database at https://microbesgenotyping.i2bc.paris-saclay.fr/databases/public (3), the Borili genotype belongs to A.Br.008/009, whereas the Baytirek genotype is assigned to the Ames/Sterne lineage. Strains KZ-100 (Borili, cattle muscle tissue), KZ-107 (Bayterek, human carbuncle), and KZ-114 (Bayterek, soil, slaughter place) were selected for sequencing. The VNTRs located on the pXO1 and pXO2 plasmids could be amplified in all the strains. Sequencing libraries were prepared using the Nextera XT DNA library prep kit (Illumina, San Diego, CA). Sequencing was performed with the MiSeq system using a MiSeq reagent kit v3 (600 cycles). The reads were trimmed using Seqtk v1.3 (5) up to value Q30 and *de novo* assembled using SKESA v2.3.0 (6). All reads were mapped back to the assembly using the Burrows-Wheeler Aligner (BWA) MEM algorithm v0.7.17 (7) to determine the average genome coverage using SAMtools v1.10 with the depth option –a (8). Single-nucleotide polymorphisms (SNPs) were identified using BioNumerics v7.6.3 (Applied-Maths, Belgium) by mapping reads on the Ames Ancestor reference (GenBank assembly accession no. GCF_000008445) and analyzed as previously described (9–11). The maximum parsimony tree was built using BioNumerics. All software was used with default parameters except when stated otherwise.

The assembly resulted in 42, 85, and 178 contigs, with *N*_50_ values of 378,397, 121,323, and 64,674 bp, total genome sizes of 5,449,226, 5,438,930, and 5,429,451 bp, average coverages of 55×, 49.5×, and 59.9×, and GC percentages of 35.1%, 35.09%, and 35.12% for B. anthracis strains KZ-100, KZ-107, and KZ-114, respectively. The presence of the pXO1 and pXO2 plasmids was confirmed by aligning the assembled contigs to reference sequences (Ames Ancestor) using BLAST+ v2.6.0 (12). Genome annotation was performed using the NCBI Prokaryotic Genome Annotation Pipeline (PGAP) v4.13 (13, 14). Totals of 5,364, 5,343, and 5,343 coding DNA sequences (CDSs) were predicted for B. anthracis strains KZ-100, KZ-107, and KZ-114, respectively.

**FIG 1:**
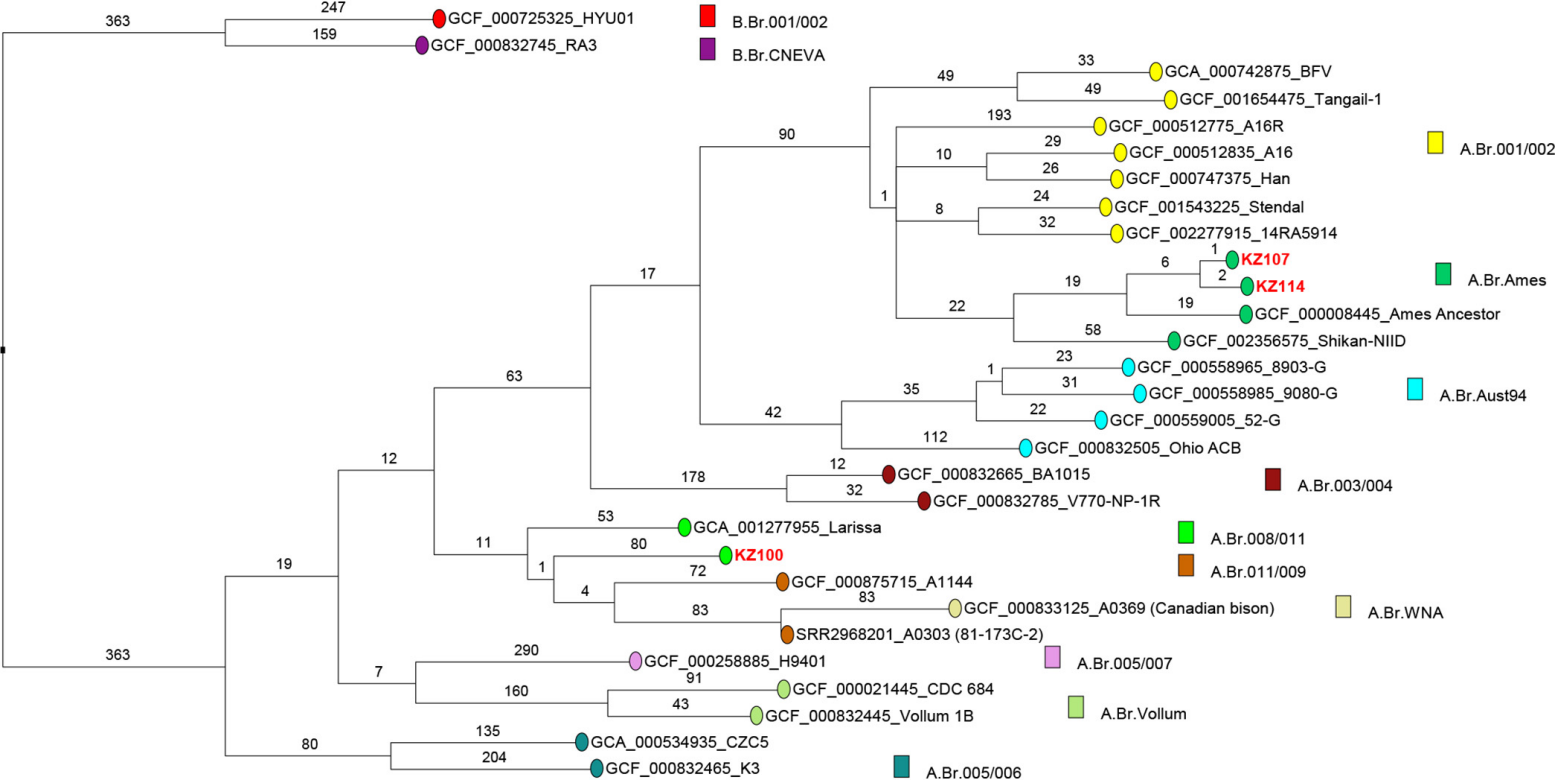
Whole-genome SNP analysis of the three strains from Kazakhstan, KZ-100, KZ-107, and KZ-114, and comparison with representative fully sequenced genomes. Twenty-six representative full genomes and three strains from Kazakhstan (bold, red type) were mapped on the Ames ancestor reference genome for SNP calling. The 3,784 SNPs identified were used to make a maximum parsimony tree. The tree size is 3,799 SNPs, corresponding to a 0.4% homoplasy level. The tree was rooted using the B branch as the outgroup. Branch lengths are shown, and assembly accession numbers and strain names are indicated. Strains are colored according to their CanSNP assignment.

Canonical SNP (CanSNP) analysis using CanSNPer v1.0.10 (15) confirmed KZ-100 as A.Br.008/011 and KZ-107/KZ-114 as A.Br.Ames. Strains B. anthracis KZ-107 and B. anthracis KZ-114 differ by three SNPs, which confirms their common source of origin (Fig. 1). To our knowledge, this is the first report of the Ames lineage in West Kazakhstan (16).

### Data availability.

This whole-genome shotgun project has been deposited in DDBJ/EMBL/GenBank under the accession no. JACTNR000000000, JACXSN000000000, and JACXSM000000000. The versions described in this paper are the first versions, JACTNR010000000, JACXSN010000000, and JACXSM010000000. The raw data from BioProject PRJNA639508 were submitted to the NCBI SRA under experiment accession no. SRR12560170, SRR12633803, and SRR12633802.
